# Acknowledgment to Reviewers of *Tomography* in 2021

**DOI:** 10.3390/tomography8010022

**Published:** 2022-01-29

**Authors:** Tomography Editorial Office

**Affiliations:** MDPI AG, St. Alban-Anlage 66, 4052 Basel, Switzerland

Rigorous peer-reviews are the basis of high-quality academic publishing. Thanks to the great efforts of our reviewers, *Tomography* was able to maintain its standards for the high quality of its published papers. Thanks to the contribution of our reviewers, in 2021, the median time to first decision was 25.5 days and the median time to publication was 55 days. The editors would like to extend their gratitude and recognition to the following reviewers for their precious time and dedication, regardless of whether the papers they reviewed were finally published:
Adam TrepczynskiJoana DamásioAgnieszka KotalczykJohn J. SunderlandAhmed OthmanJonathan MartinelliAkiko TodakaJoost Van Der VorstAkram Abdo AlmansooriJung-Woo LeeAlberto ChighineJyh-Miin LinAleš FidlerKarin Markenroth BlochAlessandro N FranciosiKarl Olof LövbladAlexander M. LongKarolina MarkietAlexandra RoudenkoKen-Ichiro MatsumotoAlexandros G. SykarasKenneth HettieAlfonso Vittorio MarchianoKeunBaDa SonAli Al KaissiKe-Vin ChangAlon BartalKevin ChenAmirmasoud AhmadiKirsten KoolstraAna FaustinoKivovics MártonAndrea AlexandreKiyotaka NemotoAndrea BorghesiKlearchos PsychogiosAndrea CassoniKo HashimotoAndrea Gonzalez-MontoroKonrad P. NesterukAndrew LavenderKuo-Chih ChenAneeza W. HamizanLechuang ChenAngela AmmirabileLina PadervinskienėAnjul KhadriaLionel Fernel GamarraAnnalisa CarlucciLubomir HadjiyskiAntonio TarasconiLuca BurroniArash NavranLuca Giovanni LocatelloAristides HatjimihailLuca PasquiniArkady SerikovLu-Han LaiArmando CavalloMajid JadidiArnaldo StanzioneMalik AydinAttila JakabManuel Durán-PovedaBarbara SpanòManuela MatesanBhupal BanMarie-Luise KromreyBo ZhaoMark J. RoefBogdana SuchorskaMartijn CloosBojan V. StimecMartina C. FussBradford A MoffatMary-Kate HaywardCaius ConstantinescuMasaki FukunagaCarlos FigueiredoMassimo BonacchiCengiz AkbulutMateusz Krystian HołdaCesare GagliardoMatteo FermiChad QuarlesMatthew F. CovingtonChengyue WuMattia ChiesaChiranjibi SitaulaMaxime RonotChristian BoozMichael GarwoodChristian SchachMichal StudniarekChristopher P. OberMichela GabelloniChristos MichailMiguel AlcarazClaus MadsenMihaela ZegreanClive KellyMikalai BudzevichCofano FabioMilagros Hidalgo De La CruzCorinne BeinatMingchung ChouCorrado AngeliniMirjam GerwingDamiani GiovanniMohamed ElsharkawyDaniel Gónzalez-BandalaMohammed R. S. SunoqrotDaniel McGowanMohd Parvez KhanDaniela MessineoMónica MartinsDaniele PironiMostafa Kamal MasudDavid Q.-H. WangMugurel Constantin RusuDeborah RundMukli PéterDiana Sorina FeierMunish ChauhanDominic GaschoNaoki OhnoDon YooNassr Al-NuaimiDonna E. GoldhawkNatalia D. GladkovaDorota Oszutowska-MazurekNektarios KoukourakisEdvard EhlerNguyen Quoc Khanh LeEdwin DickinsonNIcholas SiscoEfstratios-Stylianos PyrgelisNiels QvistEkaterina TolstayaOganes AshikyanEleonora CavallariOke GerkeEllen FunkhouserPaola Suárez-MeadeEnrico CassanoPaolo CampioniEtienne RousseauPaolo PrandoniEva HasslerPaolo SpinnatoFederico BolelliPatrycja Sosnowska-SienkiewiczFerdinand SeithPaul E. SijensFloris Van VeldenPaul MonsarratFrancesca ArezzoPaul SchoenhagenFrancesco CorattiPaul-Andrei StefanFrancesco D’OriaPaulo J. PalmaFrancesco MacriPeiman HabibollahiFrancesco RestelliPetros ChristopoulosFrancesco VerdePier Luigi StefànoFrédéric VellaPiergiorgio SolliGaurav SharmaPiero Vincenzo LippolisGautam SethiPiotr LeszczyńskiGeorg BöningPiotr MorasiewiczGerold BesserRahmeh OthmanGilbert LimRajikha RajaGina Delia Roque-TorresRalph A. BundschuhGiovanni Foti Reza PiriGuglielmo ManentiRobert Cristian PurdoiuGunnar SchuetzRobert NordstromHamza AlizaiRoddy HiramHéctor Espinós-MoratóSadiya ShaikhHenrique SilvaSebastian NagelHipolito NzwaloSelena Milicevic SephtonHiroaki NakagawaSergey Pavlovich OsipovHiroaki TobaSergio SalernoHiroshi KurokiSeungryong ChoHisham BahmadShiho NaitoHongming ShanShuiyu LuHsieh-Fu TsaiSiddharth ThakerHueisch Jy DingSneha RajuHyung-youl ParkSophie RichterIndu G. RajapakshaŠpela ŠalamonInes FasolinoSubhamoy MandalIsmini E PapageorgiouSusanne SchnellJan MejzlikSven MånssonJasper NijkampTetsuya IshikawaJee Taek KimTolga ÖzmenJelena PrpićVasileios SiokasJerzy NarlochYeou-Jiunn ChenJia Ping WuYong-An ChungJinbin XuYuki Hashimoto


